# Embossing Lines and Dots Geometry Effect on the Key Tissue Paper Properties with Finite Element Method Analysis

**DOI:** 10.3390/polym14173448

**Published:** 2022-08-24

**Authors:** Joana Costa Vieira, António de O. Mendes, Marcelo Leite Ribeiro, André Costa Vieira, Ana Margarida Carta, Paulo Torrão Fiadeiro, Ana Paula Costa

**Affiliations:** 1 Fiber Materials and Environmental Technologies Research Unit (FibEnTech-UBI), Universidade da Beira Interior, R. Marquês D’Ávila e Bolama, 6201-001 Covilhã, Portugal; 2 Aeronautical Engineering Department, São Carlos School of Engineering, University of São Paulo, Av. João Dagnone de Melo, São Carlos 13565-120, SP, Brazil; 3 Center for Mechanical and Aerospace Science and Technologies (C-MAST-UBI), Universidade da Beira Interior, R. Marquês D’Ávila e Bolama, 6201-001 Covilhã, Portugal; 4 Forest and Paper Research Institute (RAIZ), R. José Estevão, Eixo, 3800-783 Aveiro, Portugal

**Keywords:** dots and lines geometry, embossing prototype, FEM simulation, mechanical properties, softness characterization, tissue paper

## Abstract

Embossing is a functional and strategic process for creating high-quality multi-sensory tissue-paper products. Embossing modifies the sheet surface by generating hill and/or valley designs, changing the third-dimension *z* with a compressive die. This research work specifically concerns the impact study of the engraving finishing geometry on the final properties of tissue paper. This work led us to conclude that, even though the sheets individually present a higher hand-feel (HF) value for the straight finishing geometry, the highest softness was obtained in the two-ply prototype for the round finishing geometry. Moreover, this study confirmed that the HF value reduces with the increase of the bulk, being more accentuated for the micropattern. Relevant differences could not be seen in the spreading kinetics of the liquid droplets over time. Thus, the finishing geometry of the 3D plates did not impact the absorption kinetics on these samples. The finite element model allows us to understand the effect of the plate pattern and its finishing geometry on the paper, and the simulation results were in accordance with the experimental results, showing the same trend where patterns with a round finishing geometry marked the tissue-paper sheet more than patterns with a straight finishing did.

## 1. Introduction

**Figure 1:**
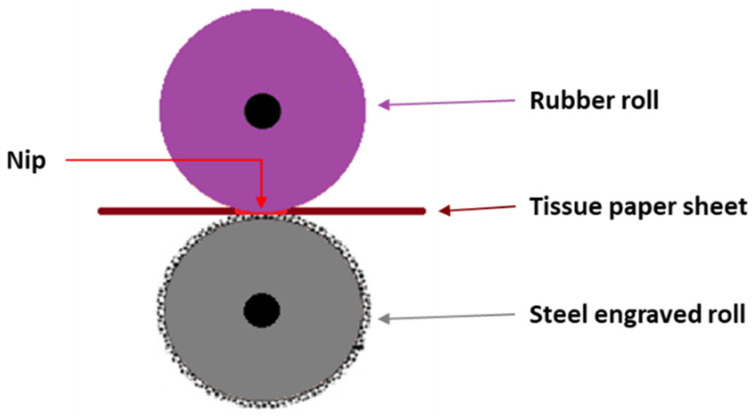
Schematic of the main performers in the tissue-paper-embossing process.

Enhancing both originality and creativity is often a brand’s selling and advertising argument. Therefore, embossing is a key process for producing high-quality multi-sensory products. Embossing is a type of compressive action that is carried out during tissue-paper converting by creating a pattern/engraving on paper with different depths, thus altering the sheet topography [1]. In addition to engrave the paper with patterns for aesthetic purposes, these hills or valleys increase the volume of the paper sheet, improving various properties, such as porosity, absorption, and softness. On the contrary, there are often losses of mechanical strength due to the damage caused to the structure of the material [2,3,4]. During the embossing process, the cellulosic fibers are compressed into well-marked permanent patterns, using pressure as force [5]. Each sheet passes through a pair of rolls, a steel roll that has the desired embossing pattern engraved on it, and often a rubber roll of a predetermined hardness. A defined pressure is applied to the nip, as presented in Figure 1, so that the engravings fulfill the intended purpose [2,3].

In theory, any kind of paper and/or cardboard can be embossed, considering the material properties, thickness, composition, fiber type, fiber length, and mechanical-strength properties (tensile strength, elongation, tear strength, delamination strength, compressive strength, and burst strength). Thus, bulkier materials will respond like a sponge, as they are more compressible, requiring higher pressure to make the engraving permanent. On the other hand, if this pressure exceeds the limit of the material, the embossing process will cause permanent damage to its structure, and this will appear on the surface of the sheet of paper [5].

A limitation of the embossing process is related to the dimensions and geometry of the embossing-pattern elements. Patterns with fine details, such as dots and fine lines, cannot be engraved to a high depth, as fine details are predisposed to tearing the sheet of paper. High-quality embossing is not only related to the engraving depth; the ability of the paper to stretch and adapt to the embosser is an equally important physical limitation [5]. There are no standardized embossing-pattern geometries and/or designs for an objective evaluation of the final properties of these structures with hills/valleys.

From the work developed by Khan [6], it was shown that different parameters of the geometry of the embossing pattern, such as the density of dots (number of dots/cm^2^), height of the dots, and the angle that the dot makes with the base, affect the deformation of the sheet and, consequently, the mechanical properties of the embossed product. Thus, Khan [6] concluded that a higher density of dots and a higher height of the dots lead to a greater deformation of the tissue-paper sheet and, consequently, to a greater loss of mechanical properties. Conversely, a higher dot angle (cylindrical dot geometry) leads to less deformation and, consequently, to a lower loss of mechanical properties. In addition to the work developed by Khan [6], the novelty of our study was keeping those parameters constant and varying the finishing geometry of the lines and dots.

The influence of embossing pressure, time, and rubber hardness on the final properties of tissue paper is known from previous works [7,8]. In the present work, we intended to study the impact of engraving geometry on the final properties of tissue paper, using four customized 3D steel plates with different finishing geometries, and to confirm the obtained results by a finite element method (FEM) simulation.

## 2. Materials and Methods

### 2.1. Materials

**Figure 2:**
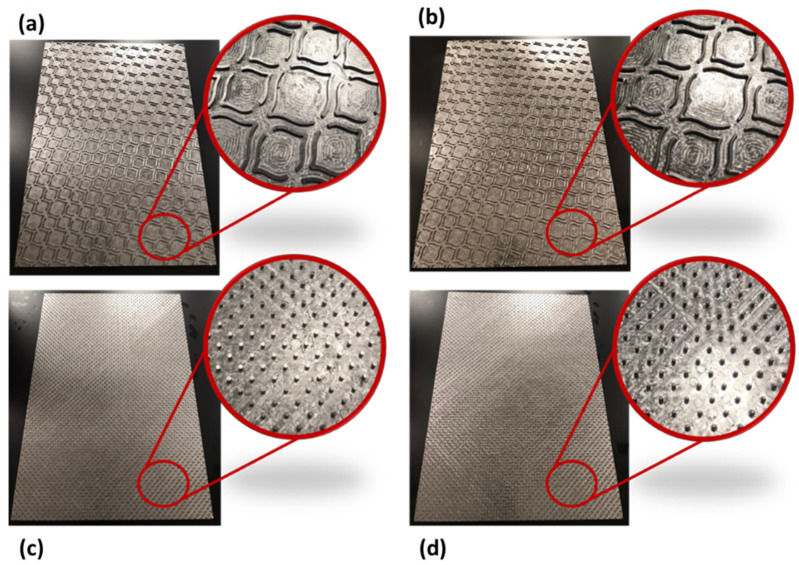
Images of the 3D steel embossing plates with the deco pattern and micropattern, and details of the respective finishing geometries: (**a**) deco pattern with straight lines, (**b**) deco pattern with round lines, (**c**) micropattern with straight dots, and (**d**) micropattern with round dots.

To carry out this work, industrial creped toilet-base tissue paper from one Portuguese factory was used. The composition of the used paper was a mixture of 30% *Eucalyptus globulus* (hardwood) and 70% *Pinus pinaster* (softwood) bleached kraft pulps. This paper, denominated by B, had a grammage of 16.7 g/m^2^ and was produced on a paper machine with a single layer headbox and with a ceramic creping blade. Moreover, for the execution of this work, four steel embossing plates were used, two of them with a deco pattern and the other two with a micropattern. Each steel plate presents dimensions of 210 × 297 mm and a different line or dot finishing geometry. In Figure 2, images of the four 3D steel embossing plates with the deco patterns and micropatterns are presented. The deco patterns were composed only by lines, and the micropatterns were composed only by dots, using both straight and round finishing geometries.

All the tissue samples are denominated according to each 3D steel-plate pattern and finishing geometry. Samples embossed with the deco pattern with straight lines are designated by adding the abbreviation d*_sl_* to the paper denomination, and those with round lines are designated by d*_rl_*. The samples embossed with the micropattern with straight dots are designated by adding the abbreviation m*_sd_* to the paper denomination, and those with round dots by m*_rd_*.

### 2.2. Methods

**Figure 3:**
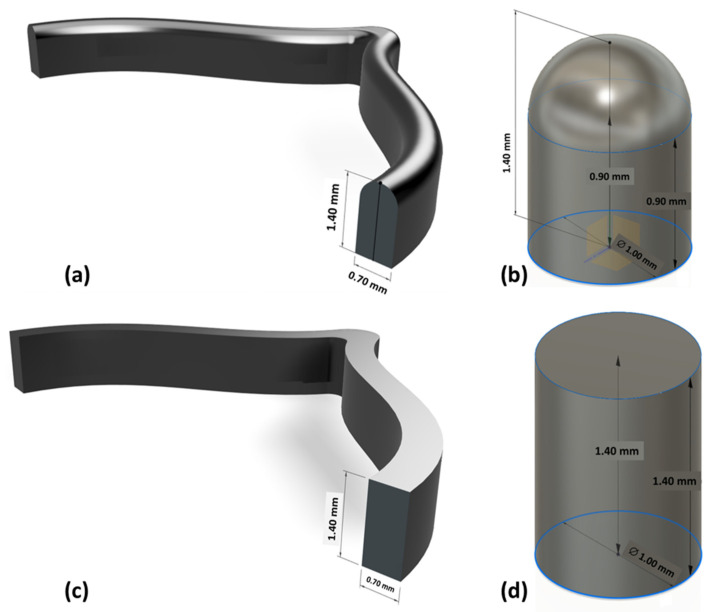
Finishing geometry and dimensions of the lines and dots engraved in the 3D steel plates: (**a**) line with a round finishing, (**b**) dot with a round finishing, (**c**) line with a straight finishing, and (**d**) dot with a straight finishing.

This work started by performing the embossing operation itself, with the four different embossing patterns engraved on steel plates. The two deco-pattern plates differ in the finishing geometry of the lines (straight or round), while the two micropattern plates differ in the finishing geometry of the dots (straight or round). Figure 3 shows the finishing geometry and dimensions of the lines and dots engraved in the 3D steel plates. The used operation conditions in the embossing process were 2.8 bar during 1 min and a variable rubber hardness of 60_48 Sh-A for the 4 types of 3D steel plates with the embossing patterns. These operating conditions were the same as those previously set by the authors in other related works [7,8].

All the tissue samples engraved with the four embossing patterns were tested in terms of thickness/bulk and their mechanical tensile strength in the machine direction (MD) and in the machine cross direction (CD). Tensile tests were performed in a Thwing-Albert^®^ VantageNX Universal testing machine, in agreement with the tissue standard ISO 12625-4:2005, and thickness and bulk were determined by using a FRANK-TPI^®^ Micrometer, fulfilling the tissue standard ISO 12625-3:2014. On all the embossed samples, the softness was tested with a Tissue Softness Analyzer (TSA) from EMTEC^®^ equipment. For the computation of the handfeel (HF), the TP II and the QA I algorithms were used.

The samples were also analyzed by using three different modules of the customized optical system for the visual inspection of their surfaces, for the topographic reconstruction of the samples in three-dimensions, and also for the study of their interaction with liquid droplets. Specifically, the first module of the optical system [9,10,11] enables the image acquisition of the surfaces of the paper samples engraved with the different embossing patterns, considering highly detailed and magnified images, having shown to be very versatile in other similar studies [12,13,14,15,16]. The second module of the optical system, originally developed for the evaluation of topographic changes manifested on the surface of textiles [17,18,19,20], was used as a complement to the first module, to further explore the inspection of the paper samples, reconstructing their surfaces in 3D, enabling us to fully observe, in different views, the differences of the engraving marks obtained with the different considered embossing plates. Recently, this module has also been used in a study that was carried out regarding the characterization of the crepe microstructure in a set of different industrial tissue samples, on which the samples used in the current study were included. Finally, the third module of the optical system [21] enables the study of the spreading kinetics of liquid droplets, performing their ejection onto the surface of the samples and acquiring images of their interaction for a given period of time (from 35.7 ms to 3 s), and, similarly as for the other two modules, this third module was also used in other conducted studies by our research team [7,22,23,24,25].

### 2.3. Finite Element Method (FEM)

The effect of the plates’ pattern geometry can be analyzed in terms of stress field and how the finishing geometry affects the plasticity after the embossing process, using the finite element models. Thus, different models were proposed to investigate in more detail these effects on the permanent deformations after the embossing process.

Since ABAQUS^TM^ does not have a native constitutive law for orthotropic plasticity material, a user material subroutine for explicit simulations (VUMAT) linked to the commercial finite element software ABAQUS^TM^ was implemented, using an orthotropic elastic–plastic material model, proposed by Mäkelä and Östlund [26]. This material model allows users to simulate the paper anisotropic behavior, since the paper response is highly dependent on fiber orientation [26]. The model assumes the decomposition of the strain tensor into an elastic strain tensor plus a plastic strain tensor (Equation (1)), while volume is conserved:

(1) $$\varepsilon_{ij}=\varepsilon_{ij}^{e}+\varepsilon_{ij}^{p}$$

where $\varepsilon_{ij}$ is the total strain, $\varepsilon_{ij}^{e}$ is the elastic strain, and $\varepsilon_{ij}^{p}$ is the plastic strain.

The VUMAT subroutine was implemented by using the concept of an isotropic plasticity equivalent [27], considering an equivalent material that correlates the orthotropic stress state to an equivalent isotropic stress state. The relation between the actual Cauchy stress tensor and the isotropic plasticity equivalent (IPE) deviatoric tensor is given in Equation (2):

(2) $$s_{ij}=L_{ijkl}\sigma_{kl}$$

where $s_{ij}$ is the deviatoric IPE stress tensor, $\sigma_{kl}$ is the Cauchy stress, and $L_{ijkl}$ is the fourth-order transformation tensor shown in Equation (3) for plane stress:

(3) $$L=\left[\begin{array}{ccc} 2A & C-A-B & 0\\ C-A-B & 2B & 0\\ B-C-A & A-B-C & 0\\ 0 & 0 & 3D \end{array}\right]\;$$

where the parameters *A*, *B*, *C*, and *D* were calibrated from tensile-test results by using the following Equations (4)–(11) [26]:

(4) $$A=\sqrt{1-12x^{2}}\;$$

(5) B=3(y−x) 

(6) C=3(y+x) 

(7) $$D=\frac{K_{12}^{\frac{n}{\left(n+1\right)}}}{\sqrt{3}}\;$$

(8) $$x=\sqrt{\frac{\alpha^{2}}{24\left(3\alpha^{2}+\beta^{2}-4\beta +4\right)}\left(\beta +1-\sqrt{6\beta -3\alpha^{2}-3}\right)}\;$$

(9) $$y=\frac{\alpha }{4x}-A\;$$

(10) $$\alpha =K_{33}^{\frac{2n}{\left(n+1\right)}}-K_{22}^{\frac{2n}{\left(n+1\right)}}\;$$

(11) $$\beta =K_{33}^{\frac{2n}{\left(n+1\right)}}+K_{22}^{\frac{2n}{\left(n+1\right)}}\;$$

The parameters $K_{ii}$ and n were obtained by inverse parametrization, using the Ramberg–Osgood methodology to minimize the error between the tensile tests results and the model results. For the MD tensile test (Equation (12)):

(12) $$\varepsilon_{11}=\frac{\sigma_{11}}{E_{11}}+{\left(\frac{\sigma_{11}}{E_{0}}\right)}^{n}$$

For the CD, the equation is as follows (Equation (13)):

(13) $$\varepsilon_{kk}=\frac{\sigma_{kk}}{E_{kk}}+{\left(\frac{K_{kk}E_{kk}}{E_{0}}\right)}^{n},k=2,3$$

Note that, for Equation (13), the repeated indices do not mean the usual summation rule used in the indicial notation. Finally, the parameter $K_{12}$ was obtained by using Equation (14):

(14) $$\gamma_{12}=\frac{\sigma_{12}}{G_{12}}+{\left(\frac{K_{12}\sigma_{12}}{E_{0}}\right)}^{n}$$

In the case of this study, it is possible to consider *K*_22_ = *K*_33_, since the mechanical behavior in CD (direction 2) is similar to that on the thickness direction (direction 3). Thus, *A* = 1 for all models. For deco models, *B* = 2.40, *C* = 2.40, and *D* = 1.38, and for micro models, *B* = 2.44, *C* = 2.44, and *D* = 1.40. Since the tensile vs. strain curves had a similar shape, those parameters are almost the same in both MD (direction 1) and CD (direction 2).

The Hooke’s law for plane stress, small strain, and linear elastic orthotropic material is given by using Equation (15):

(15) $$\sigma =C:\varepsilon^{e}$$

where σ is the second-order Cauchy stress tensor; C is the four-order plane stress, linear elastic, orthotropic constitutive law tensor; and $\varepsilon^{e}$ is the second-order small strain elastic tensor.

The implementation of this model is similar to the well-known $J_{2}$ flow theory for isotropic materials, using the backward-Euler algorithm. The explicit solver was used to overcome convergence issues that are usual when using the implicit solver for this type of simulations. On the other hand, the stable time increment is very small, which increases the computational costs. Simulations were performed by using a workstation with two intel Xeon E5-2630 8 cores (16 cores total with 32 threads) with 256 Gb ram.

**Figure 4:**
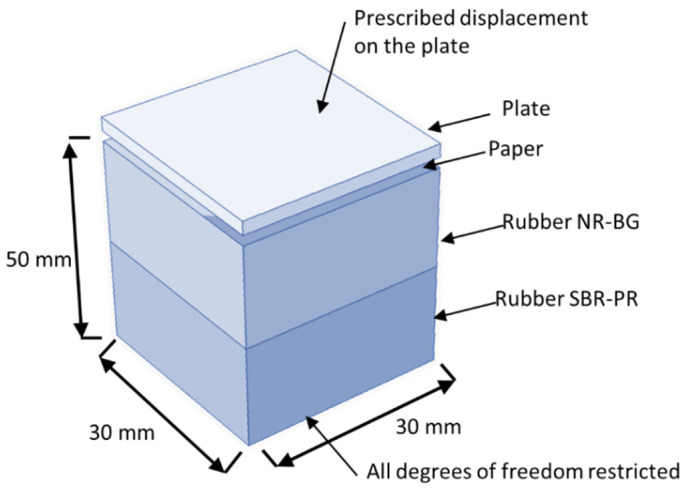
Model dimensions, characteristics, and boundary conditions.

The finite-element-model dimensions and boundary conditions are presented in Figure 4. The plate model dimensions are representative of the actual plate, resulting in a reasonable computational cost and accuracy. The rubber base dimensions were the same, but it was thick enough to allow the deformation without severe interference in the plate and paper kinematics. The paper had the same dimensions of the die and basis.

The boundary conditions were imposed to represent the embossing process. Thus, all the displacements’ degrees of freedom were restricted in the bottom of the rubber base, and a prescribed displacement was applied on the top of the plate, corresponding to a manufacture pressure of 2.8 bar applied during the embossing process. To model the contact interactions between each part of the model, hard contact was considered for normal behavior, and the tangential behavior was modeled by using penalty interaction with 0.3 for friction coefficient.

The model has 473,694 20-node hexahedral elements (C3D20) for the simulation of the micro die with round finishing dots, and 476,153 20-nodes hexahedral (C3D20) elements for the deco die with round finishing lines. To improve the simulation, the straight finishing dots of the micro die was modeled with 443,206 10-node tetrahedral mixed-mode elements (C3D10M), and 1,491,454 10-node tetrahedral mixed-mode elements (C3D10M) for the straight finishing lines of the deco die.

For all models, paper was simulated by using a 4-node reduced integration membrane element (M3D4R). The structured mesh has a total of 250,000 elements. Finally, the rubber base was modeled by using a total of 216,000 8-node hexahedral elements (C3D8).

This model uses 3 different types of materials: steel for the die (E = 200 GPa, μ = 0.33); a hyper-elastic isotropic material model for the rubber basis, SBR-PR (C_10_ = 0.810343 MPa and D_1_ = 0.56956 MPa^−1^), and for NR-BG (C_10_ = 0.409852 MPa and D_1_ = 1.126109 MPa^−1^). Paper was modeled as a user orthotropic material (E_11_ = 13.89 MPa, E_22_ = E_33_ = 4.23 MPa, μ = 0.33, and G_12_ = 2.1 MPa). As mentioned above, the parameters B, C, and D for the IPE model consider the different mechanical behavior for the deco pattern and micropattern.

The finite element model does not consider a compression force on the paper, since a plane stress constitutive relation was adopted in the used membrane element. On the other hand, the material parameters used for the simulations of the other materials (rubbers and dies) consider a manufacture pressure of 2.8 bar applied during the embossing process.

## 3. Results

**Figure 5:**
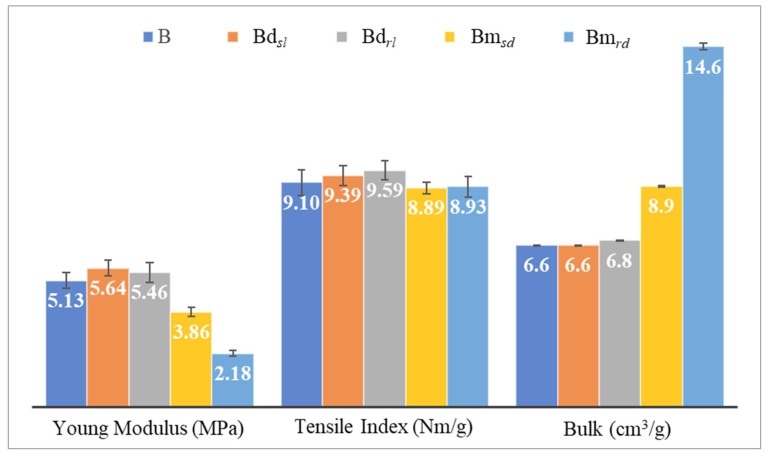
Results of mechanical characterization and bulk obtained for all the embossed samples and the reference paper, B, for the different finishing geometries of the 3D steel plates.

This work began with the mechanical characterization and determination of the bulk of the previously embossed samples and the industrial base tissue paper. The results obtained are shown in Figure 5.

**Figure 6:**
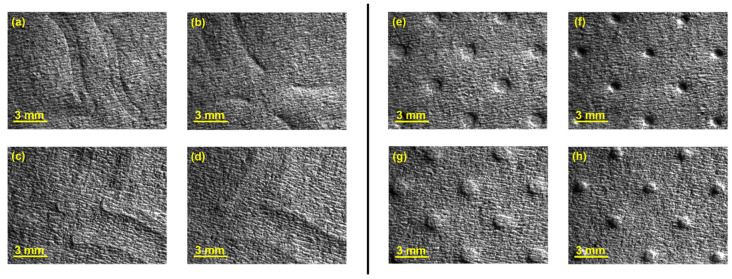
Global views of the tissue-paper samples engraved with the different considered embossing plates: (**a**) Bd*_sl_* (front side), (**b**) Bd*_rl_* (front side), (**c**) Bd*_sl_* (back side), (**d**) Bd*_rl_* (back side), (**e**) Bm*_sd_* (front side), (**f**) Bm*_rd_* (front side), (**g**) Bm*_sd_* (back side), and (**h**) Bm*_rd_* (back side).

The images obtained by analysis with the first module of the optical system of the tissue samples embossed with the four different 3D steel plates are presented in Figure 6.

**Figure 7:**
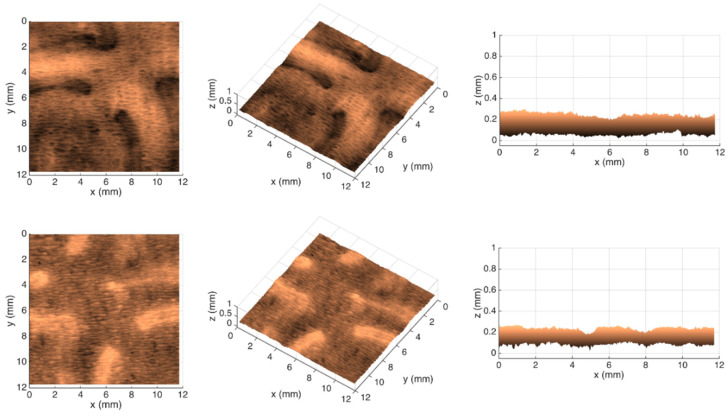
Different views of the 3D maps created for the front and back sides of the tissue-paper sample Bd*_sl_*.

**Figure 8:**
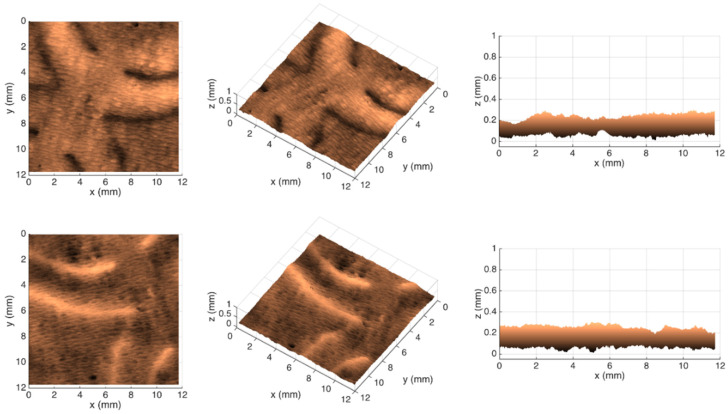
Different views of the 3D maps created for the front and back sides of the tissue-paper sample Bd*_rl_*.

**Figure 9:**
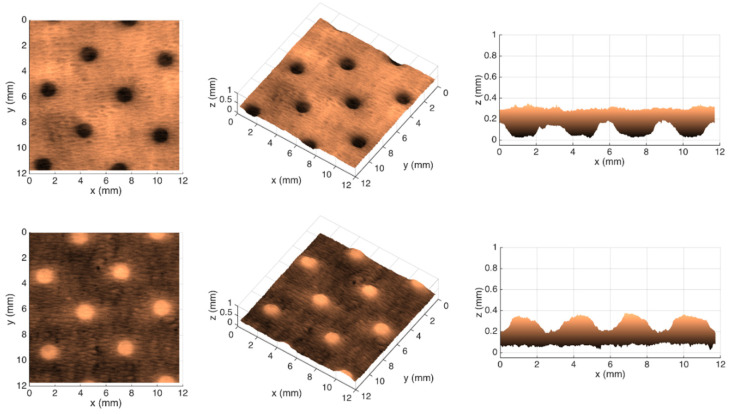
Different views of the 3D maps created for the front and back sides of the tissue-paper sample Bm*_sd_*.

**Figure 10:**
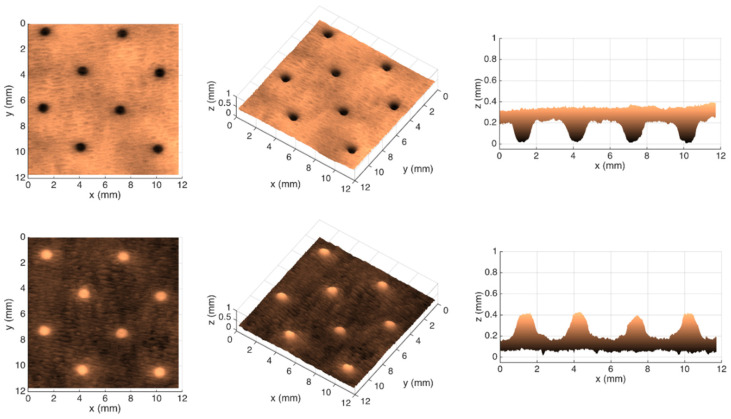
Different views of the 3D maps created for the front and back sides of the tissue-paper sample Bm*_rd_*.

Figure 7, Figure 8, Figure 9 and Figure 10 show the 3D maps created for each one of the studied cases, using the second module of the optical system.

**Figure 11:**
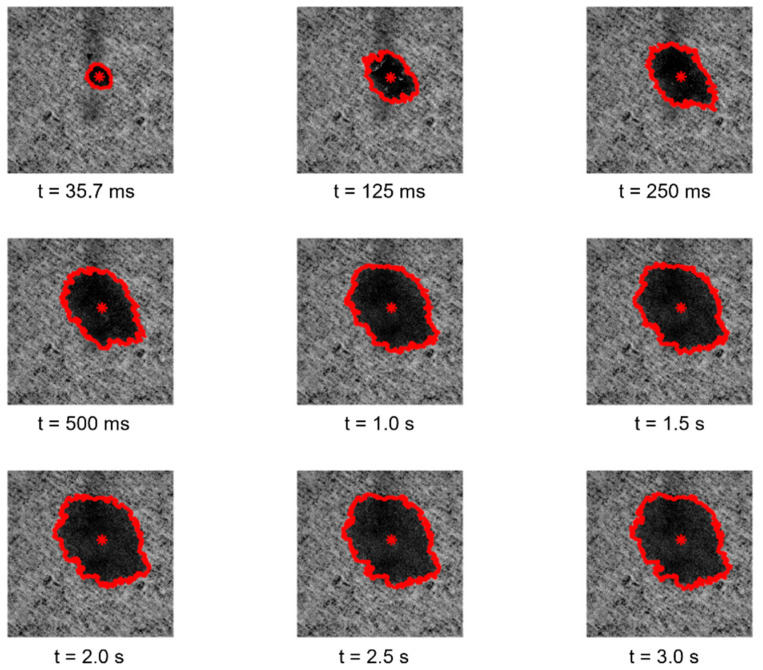
Spreading dynamics of a liquid droplet from t = 35.7 ms to 3 s for the tissue-paper sample Bd*_sl_*.

**Figure 12:**
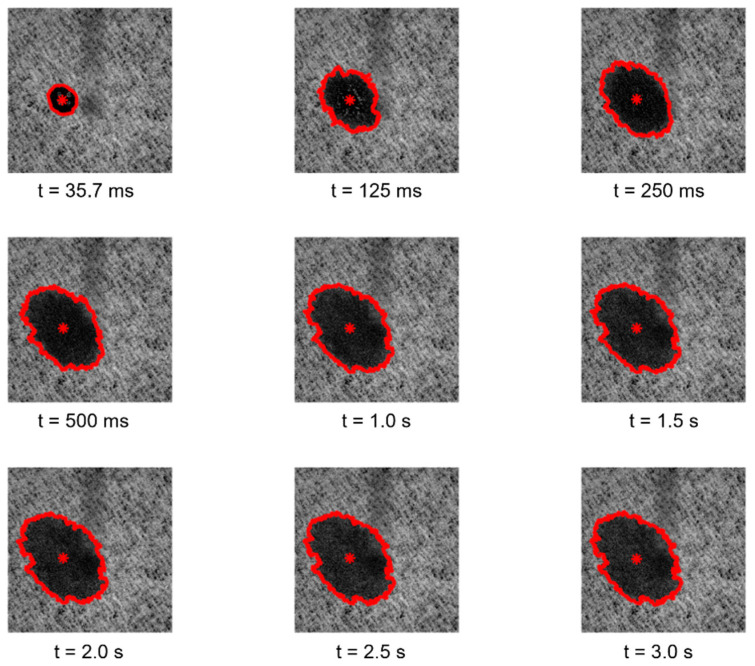
Spreading dynamics of a liquid droplet from t = 35.7 ms to 3 s for the tissue-paper sample Bd*_rl_*.

**Figure 13:**
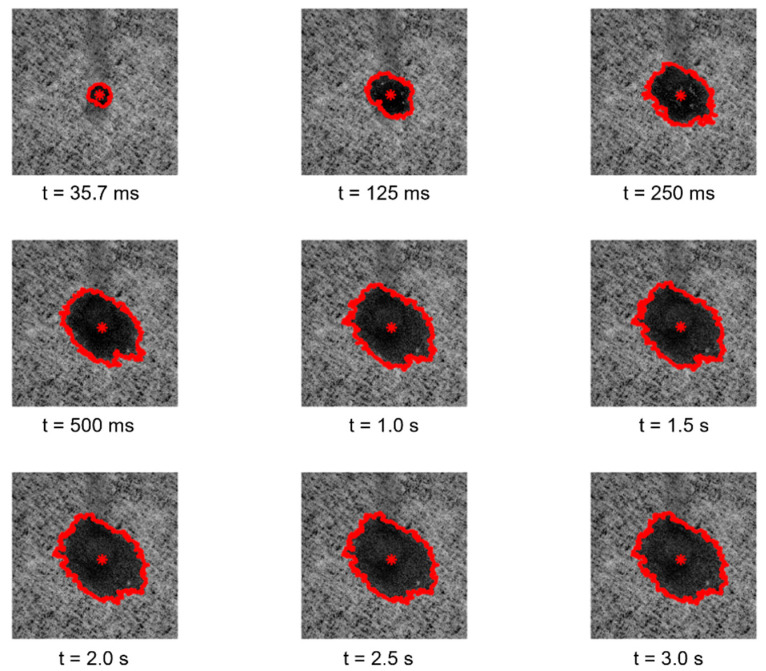
Spreading dynamics of a liquid droplet from t = 35.7 ms to 3 s for the tissue-paper sample Bm*_sd_*.

**Figure 14:**
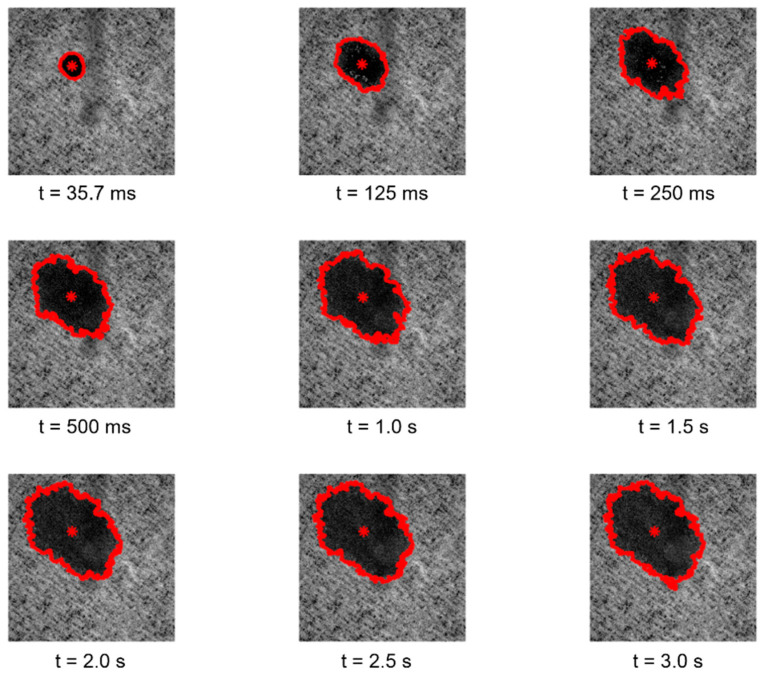
Spreading dynamics of a liquid droplet from t = 35.7 ms to 3 s for the tissue-paper sample Bm*_rd_*.

**Figure 15:**
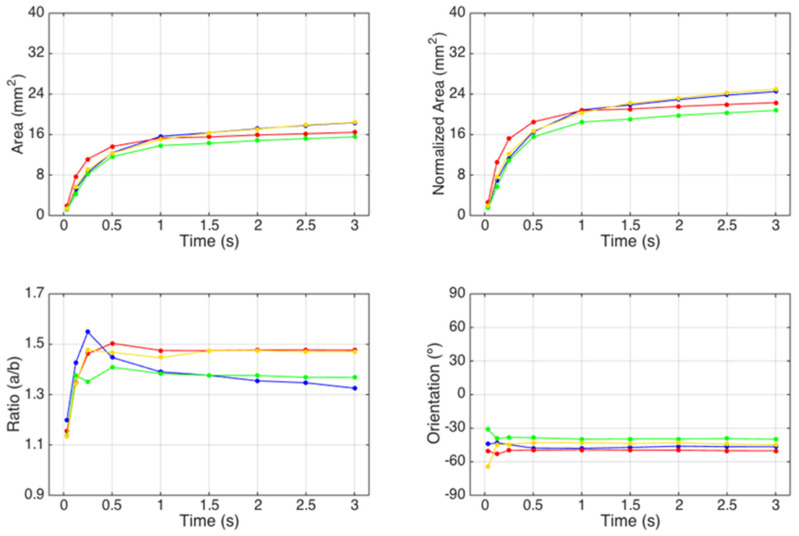
Comparative graphs of the spreading dynamics of a liquid droplet for the tissue-paper samples Bd*_sl_* (in blue), Bd*_rl_* (in red), Bm*_sd_* (in green), and Bm*_rd_* (in yellow).

Regarding the essays conducted with the third module of the optical system, the images and the results obtained for the study of the spreading dynamics of a liquid droplet on the surface of the tissue-paper samples engraved with the different embossing plates can be seen in Figure 11, Figure 12, Figure 13, Figure 14 and Figure 15.

**Figure 16:**
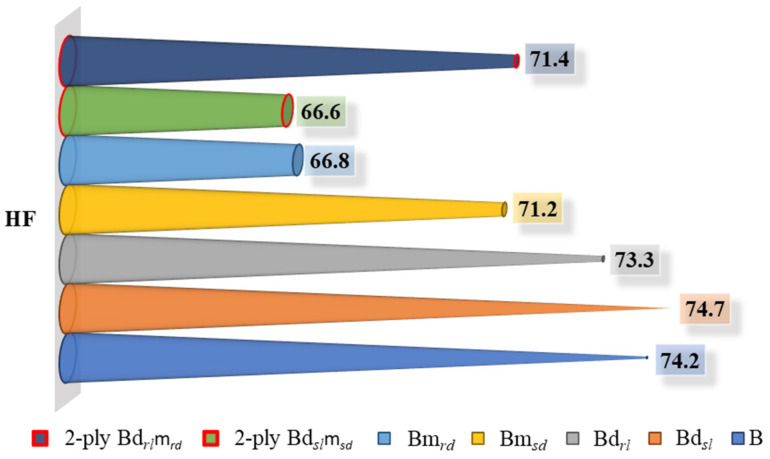
Results obtained for handfeel (HF) for all the embossed samples and the reference paper, B, for the different finishing geometries of the 3D steel plates.

By combining the deco and micro embossed sheets for each finishing geometry, straight or round, a prototype of a two-ply finished product was obtained. With the aim of optimizing the softness as a function of the 3D steel-plate finishing geometry in the embossing process, the handfeel (HF) of each one of these prototypes was measured, and the results are shown in Figure 16.

**Figure 17:**
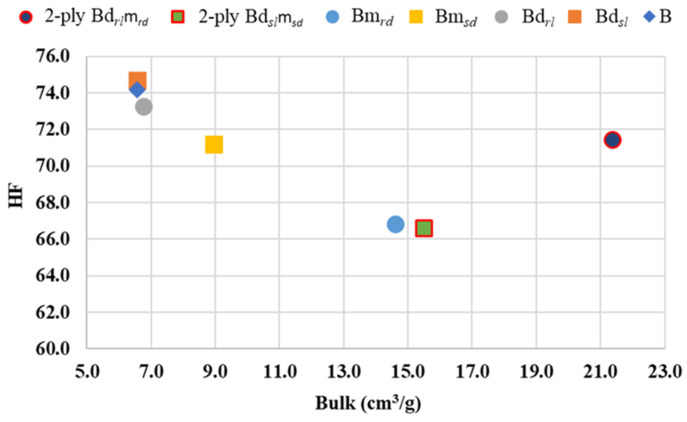
Handfeel (HF) behavior as a function of bulk.

In order to better understand the effect of embossing on softness, Figure 17 shows the behavior of handfeel (HF) as a function of bulk.

### Finite Element Method (FEM)

**Figure 18:**
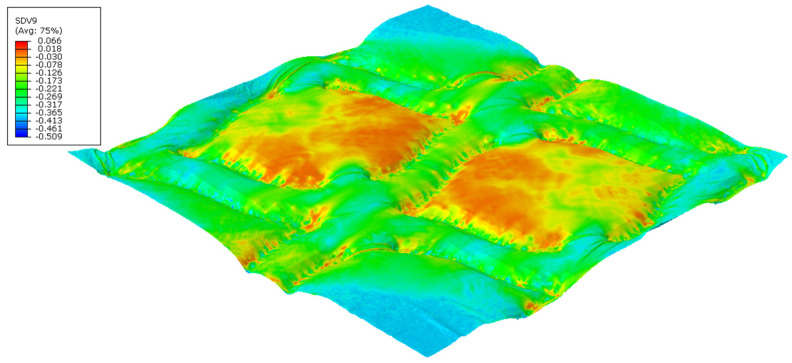
Plastic-stress-field finite-element results for deco pattern with round finishing.

**Figure 19:**
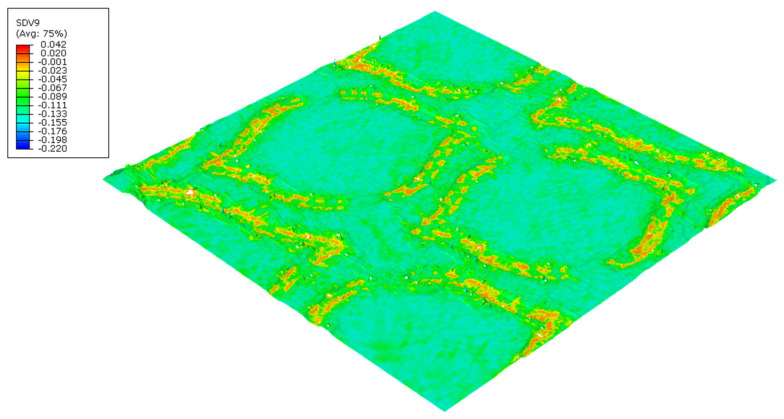
Plastic-stress-field finite-element results for deco pattern with straight finishing.

**Figure 20:**
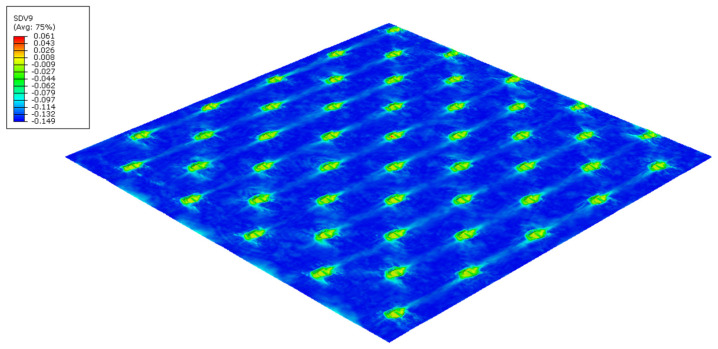
Plastic-stress-field finite-element results for micropattern with round finishing.

**Figure 21:**
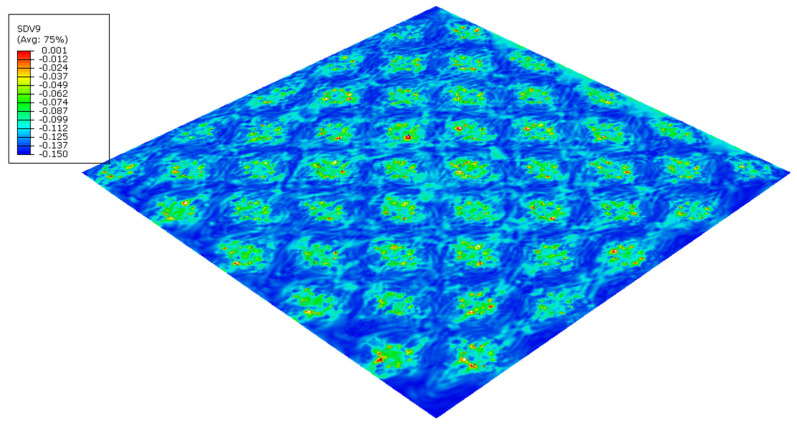
Plastic-stress-field finite-element results for micropattern with straight finishing.

The finite element models can predict the effect of the die finishing geometry on the paper. Figure 18 and Figure 19 shows the plastic stress field (*J*_2_ invariant) model results for the round- and straight-line finishing of the deco pattern, respectively, as well as the deformed shape, and Figure 20 and Figure 21 shows the results for the round- and straight-dot finishing for the micro die.

## 4. Discussion

Looking at Figure 5, in terms of sheet rigidity, we see that the micropattern affects this property of the sheet more than the deco pattern. Within each pattern (deco and micro) the round finishing geometry is the one that impacts the structure of the sheet the most. On the other hand, and bearing in mind that the embossing was carried out at the optimum pressure of the embossing system [7,8], when compared to the industrial base tissue paper, the losses and gains in mechanical strength are not very excessive, being within the standard deviation, despite, and as expected, the micropattern presenting the highest loss of mechanical strength. In both embossing patterns, the straight finishing geometry is the one with the greatest loss of this property. Moreover, as expected and in line with the results obtained for the mechanical properties, the bulk is quite superior in the micropattern when compared to the deco pattern. In terms of the line and dot finishing geometry of the embossing patterns, the round finishing stands out again, doubling the bulk value when compared to the industrial base tissue paper.

From the images shown in Figure 6, it can be seen that, in fact, the micropatterns affect the surface of the tissue-paper samples more extensively than the deco patterns do. It can also be seen from the images that the marks engraved with the deco pattern and micropattern with straight finishing appear larger than the marks engraved with the deco pattern and micropattern with round finishing. However, the marks engraved with the round finishing geometry appear to be deeper than the first ones. This can also be observed in Figure 7, Figure 8, Figure 9 and Figure 10, which show the 3D maps created for each one of the studied cases, using the second module of the optical system. The 3D maps confirm that the marks (lines and dots) engraved with the straight finishing geometry are larger in area than the marks engraved with the round finishing geometry. They also confirm that the same marks are deeper for the engravings performed with the round comparatively to the straight finishing geometries. This is very clear by observation of the above figures, especially for the micropatterns. This means that the steel plates that were manufactured with the round finishing geometry, because of their characteristic design, provide a higher penetration of the engravings in the paper structure. Therefore, they provide a higher impact in the depth of the paper sheets, especially in the paper sample engraved with the micropattern, on which the marks are the deepest, with the highest values in the z direction.

By observing the images seen in the Figure 11, Figure 12, Figure 13 and Figure 14, it can be noticed that there are no relevant differences in the spreading of the liquid droplets over time, when comparing the four considered cases. In particular, it can be seen that the droplets spread wider over time, and then they tend to stabilize around t = 1.0 to 1.5 s. In all cases, the droplets assume elliptical shapes, following the creping lines present in the surface of the tissue samples that are aligned approximately with the diagonal of the images. Globally, the areas occupied by the droplets and their shapes are very similar in the four cases. The comparative graphs shown in the Figure 15 confirm exactly the same, revealing evolutions over time very close and in the same range of values in all the considered cases for the parameters “Area”, “Normalized Area”, and “Orientation”. The only parameter that shows some differences is the “Ratio” of the shape assumed by the droplets, as it was revealed to be more elliptical for the tissue-paper sample engraved with the deco pattern with round finishing (in red) and for the tissue-paper sample engraved with the micropattern with round finishing (in yellow). The other two cases, namely the tissue-paper sample engraved with the deco pattern with straight finishing (in blue) and the tissue-paper sample engraved with the micropattern with straight finishing (in green), revealed lower values of ellipticity; however, they still show very pronounced elliptical shapes (see images of Figure 11, Figure 12, Figure 13 and Figure 14). From the images and the third graph of Figure 15, it can also be noted that there is a high peak that appeared for the instant of time t = 250 ms for the case of the tissue-paper sample engraved with the deco pattern with straight finishing (in blue). In terms of the “Orientation” of the droplets spreading (fourth graph of Figure 15), it can be seen that all cases are contained in the same range, as already stated earlier, coincident with the diagonal of the images (approximately −45°), which is the angle at which the creping lines are seen. In sum, the results obtained for the spreading dynamics of liquid droplets have shown to be very similar for all the considered cases, meaning that the finishing geometry of the elements contained in the steel plates did not have a significant impact on the assays conducted with the third optical system.

By analyzing Figure 16 and looking at the finishing geometry of the lines and dots individually, we can see that, for the two embossing patterns, the straight finish of the 3D steel plates results in a higher HF value. This result is mainly associated with the low effect of the surface roughness of the sheet, very close to the non-embossed industrial base tissue paper. On the other hand, when we analyze the two-ply prototypes, it is possible to verify that this trend is reversed, with the prototype with a round finish having the highest HF value. Observing Figure 17 and considering the sheets individually embossed with the 3D plates of steel, it is verified that, with the increase of the bulk, there is a decrease of the HF, and this decrease is more accentuated for the micropattern. Looking at the two-ply prototypes, and as mentioned before, we can see that the prototype with a round finish is the one that has the highest HF value, and this happens due to the contribution of bulk softness. In a two-ply prototype, the HF is measured at the top surface, where the deco pattern embossing is found. This pattern, as seen, is the one that least affects the softness, so the parameter that is contributing to the increase in the HF value is the bulk. Thus, to optimize the final softness of the toilet paper, the round finishing of the deco and micro embossing patterns would be the best choice for the producer.

Figure 18, Figure 19, Figure 20 and Figure 21 show the amount of plasticity obtained in the paper, using these models, due to the embossing process. In these figures, it is possible to observe the regions where the plastic deformations were detected and their distribution over the paper’s surface. When we compared Figure 18 and Figure 19, for the deco pattern, the plastic stress field for the round finishing showed that the plastic stress field is more spread over the paper’s surface (Figure 18), while for the straight finishing, the plastic stress field is concentrated in the regions near the edges, where the peaks of the plate touch the paper (Figure 19). On the other hand, when we compared Figure 20 and Figure 21, for the micropattern, we noted that the plastic stress field for the round finishing is concentrated in the regions where the die peaks touch the paper (Figure 20), while the plastic stress field for the straight finishing is more spread over the paper surface (Figure 21). Hence, the simulations enable us to show that a round finishing produces a concentrated plastic-stress-field distribution over the paper’s surface, as well as higher stresses, in accordance with experimental results where the round finishing produces a more marked pattern compared to the straight finishing. This trend is observed in both deco patterns and micropatterns. The simulation of the embossing process shows that the finishing of the patterns influences the plastic-stress-field distribution, more or less concentrated; the maximum stress; and the regions where higher stress values occur. For the plates with straight finishing, the plastic field is more pronounced on the top of the pattern along the contact edge, but with the plates with round finishing, the plastic field is more pronounced on the base contact region of the pattern.

## 5. Conclusions

The study of the impact of the finishing geometry of the line and dots (straight or round) of steel 3D plates in the embossing process led us to conclude that, although the sheets individually present a higher HF value for straight finishing geometry, in the two-ply prototype, the highest softness was obtained for the prototype with the round finishing geometry. Moreover, for the sheets embossed individually, it was verified that the HF value decreases with the increase of the bulk, and this was more accentuated for the micropattern.

It was also concluded that there are no relevant differences in the spreading kinetics of the liquid droplets over time. Thus, the finishing geometry of the steel 3D plates does not affect the absorption kinetics of these products.

The finite element model allowed to understand the effect of the plate patterns’ finishing geometry on the paper due to the embossing process changing the plastic stress field. The simulation results were in accordance with the experimental results. These were able to show a trend where patterns with round geometry marked the tissue-paper sheet more than patterns with straight finishing did. This increase of the marks corresponds to an increase of bulk. Furthermore, this tool enables the optimization of process parameters; —in this work, particularly in regard to the pattern’s finishing geometry—in a virtual environment, thus avoiding a costly trial–error approach.

This study highlighted the importance of the patterns’ finishing geometry and its impact on the final properties of tissue-paper products. The authors consider that the obtained results contribute to creating a better understanding of the embossing process when analyzing the impact of each embossing operation condition separately—in this case, the finishing geometry of the lines and dots in the finish of steel 3D plates.

## Data Availability

Not applicable.
